# A generalizable and open-source algorithm for real-life monitoring of tremor in Parkinson’s disease

**DOI:** 10.1038/s41531-025-01056-2

**Published:** 2025-07-10

**Authors:** Nienke A. Timmermans, Roberta Terranova, Diogo C. Soriano, Hayriye Cagnan, Yordan P. Raykov, Ioan Gabriel Bucur, Bastiaan R. Bloem, Rick C. Helmich, Luc J. W. Evers

**Affiliations:** 1 Department of Neurology, Center of Expertise for Parkinson and Movement Disorders, Radboud University Medical Center, Institute for Brain, Cognition and Behavior, Nijmegen, The Netherlands; 2 Department of Medical, Surgical Sciences and Advanced Technologies G.F. Ingrassia, University of Catania, Catania, Italy; 3 MRC Brain Network Dynamics Unit, Nuffield Department of Clinical Neurosciences, University of Oxford, Oxford, UK; 4 Federal University of ABC, São Bernado do Campo, Brazil; 5 Department of Bioengineering, Imperial College London, London, UK; 6 School of Mathematical Sciences, University of Nottingham, Nottingham, UK; 7 Institute for Computing and Information Sciences, Radboud University, Nijmegen, The Netherlands

**Keywords:** Parkinson's disease, Databases

## Abstract

Wearable sensors can objectively and continuously monitor daily-life tremor in Parkinson’s Disease (PD). We developed an open-source algorithm for real-life monitoring of PD tremor which achieves generalizable performance across different wrist-worn devices. We achieved this using a unique combination of two independent, complementary datasets. The first was a small, but extensively video-labeled gyroscope dataset collected during unscripted activities at home (*n* = 24 PD; *n* = 24 controls). We used this to train and validate a logistic regression tremor detector based on cepstral coefficients. The second was a large, unsupervised dataset (*n* = 517 PD; *n* = 50 controls, data collected for 2 weeks with a different device), used to externally validate the algorithm. Results show that our algorithm can reliably quantify real-life PD tremor (sensitivity of 0.61 (0.20) and specificity of 0.97 (0.05)). Weekly aggregated tremor time and power showed excellent test-retest reliability and moderate correlation to MDS-UPDRS rest tremor scores. This opens possibilities to support clinical trials and individual tremor management with wearable technology.

## Introduction

Parkinson’s Disease (PD) is the fastest-growing neurodegenerative disease with a prevalence of 11.8 million people worldwide in 2021^1^. There is currently no cure for PD, but treatments are available to alleviate symptoms. Tremor is one of the cardinal motor symptoms of PD, occurring in approximately 75% of persons with PD^2,3^. It is experienced as one of the most bothersome symptoms, especially in early disease stages^4,5^. The typical Parkinsonian tremor is a 3-7 Hz rest tremor in the upper limb, although it may also occur in the lower limb or jaw^6,7^. Re-emergent tremor, pure postural tremor and kinetic tremor are also frequently observed^6^.

Adequate assessment of tremor severity and its context-dependency could allow for more personalized treatment, and more efficient clinical trials evaluating new treatments^3,8^. However, the highly variable expression of tremor complicates its evaluation during the typically brief, episodic clinical visits^4^. In many patients, the expression of tremor is influenced by emotional and cognitive stress, voluntary movements, and timing of treatments^9^. Furthermore, the currently used Movement Disorder Society - Unified Parkinson’s Disease Rating Scale (MDS-UPDRS) for clinical tremor assessment is limited by inter- and intra-rater variability^10^. A home diary is another tool for tremor assessment, but suffers from poor compliance and recall bias^11^.

These limitations could be overcome by using wearable sensors that continuously and objectively measure tremor presence and severity in daily life^12,13^. Several algorithms have been developed for remote monitoring of tremor in PD, mostly using a wrist-worn accelerometer, gyroscope, or a combination thereof^14–26^. However, these wearable sensors and algorithms have not yet been introduced widely into clinical practice and research^12,13,27,28^.

Two critical aspects must be addressed to enable robust monitoring of tremor in daily life. First, training of supervised machine learning algorithms relies on accurately labeled datasets based on concurrent video recordings, which are often collected in highly controlled environments^26^. However, algorithms trained on these datasets are usually not generalizable to data collected in real life^29^. Second, there is a need for open-source algorithms with generalizable performance across different sensor devices and across study populations^30,31^. This would facilitate its adoption in clinical trials and care, and facilitate bring-your-own-device solutions. However, external validation of tremor detection algorithms on datasets using different devices is often lacking, despite potential performance degradation due to varying sensor positions or sensitivity to noise^21,32^.

Here, we address these issues by developing and validating an open-source algorithm for real-life monitoring of rest tremor in PD. The algorithm is trained on video-labeled data collected with a wrist-worn gyroscope sensor, during unscripted daily life activities (Parkinson@Home Validation Study)^33^. We subsequently applied the algorithm to free-living, unsupervised data collected during the Personalized Parkinson Project (PPP) using another wrist-worn gyroscope sensor^34^. Using this dataset, we assessed the generalizability of the developed algorithm. Finally, we determined the construct validity and test-retest reliability of several sensor-derived weekly aggregated tremor measures.

## Results

**Fig. 1:**
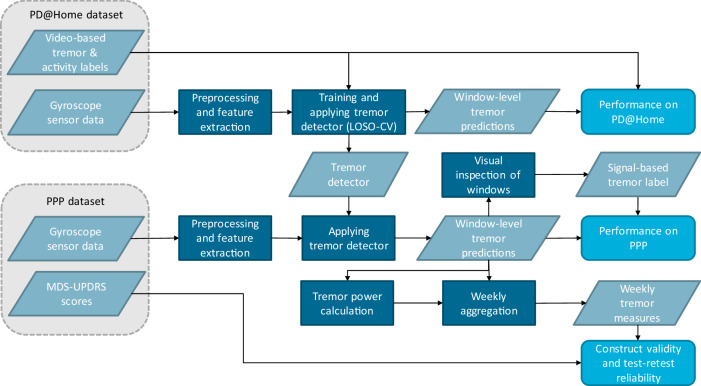
Overview of the development and validation of the tremor detection algorithm using the PD@Home and PPP datasets. The tremor detector was trained and applied to obtain window-level tremor predictions, which were subsequently aggregated to obtain weekly tremor measures. LOSO-CV leave-one-subject-out cross-validation.

Figure 1 shows a high-level overview of the steps taken to develop and validate the tremor detection algorithm. The performance of the algorithm on the PD@Home and PPP datasets will be described below, as well as the construct validity and test-retest reliability of weekly aggregated tremor measures.

### Tremor detection performance on PD@Home

Details of the tremor detection algorithm can be found in the methods section. Briefly, we trained a logistic regression classifier to detect tremor based on scale-insensitive mel-frequency cepstral coefficients (MFCCs) that capture the periodicity in wrist gyroscope signals. The first five MFCCs contributed most to the classifier (Supplementary Fig. 1), which capture slower variations across the frequency spectrum of the signals compared to the higher-order MFCCs^35^. After applying the logistic regression classifier, we filtered out detected tremor windows with a frequency peak outside the rest tremor range of 3-7 Hz^7^, since we aimed for a high specificity for rest and re-emergent tremor. We also filtered out all windows with detected non-tremor arm movements to further increase the specificity for rest tremor.

Table 1 shows the sensitivity and specificity of the tremor detection algorithm on the PD@Home dataset, for different subgroups and types of activities. The specificity in the tremor PD group was lower than in the non-tremor PD and non-PD control groups, and this difference remained after correcting for differences in the prevalence of the four activities that all subjects performed (sitting, standing, gait and postural transitions). Filtering out windows with detected non-tremor arm movements (e.g. arm swing during gait) increased the specificity for tremor from 96% to 97%, corresponding to a 25% reduction in false positives (see Supplementary Table 1 for the performance without filtering out windows with detected non-tremor arm movements). Although this comes at the cost of a slightly decreased overall sensitivity, it reduces the number of false positive tremor windows when measuring over a longer time period where often the non-tremor class is more prevalent.

**Table 1:** Tremor detection performance on PD@Home (average across leave-one-subject out cross-validation folds, and standard deviation between brackets), for different subgroups and different types of activities

	Sensitivity	Specificity	Weighted specificity across sitting, standing, gait and postural transitions
Overall (*n* = 48)	0.61 (0.20)	0.97 (0.05)	
**Subgroup**			
Tremor PD (*n* = 8)	0.61 (0.20)	0.91 (0.04)	0.92 (0.05)
Non-tremor PD (*n* = 16)	–	0.96 (0.06)	0.97 (0.05)
Non-PD controls (*n* = 24)	–	0.99 (0.01)	0.99 (0.01)
**Type of activity**			
Sitting (*n* = 8/*n* = 48)	0.62 (0.28)	0.96 (0.07)	
Standing (*n* = 7/*n* = 48)	0.48 (0.34)	0.99 (0.03)	
Gait (*n* = 7/*n* = 48)	0.01 (0.03)	1 (0)	
Postural transitions (*n* = 48)	-	1 (0.01)	
Running/Exercising (*n* = 5)	-	1 (0)	
Cycling (*n* = 14)	-	1 (0)	
Driving motorized vehicle (*n* = 2)	-	1 (0.01)	
Significant upper limb activity (*n* = 8)	-	0.98 (0.03)	
Periodic activities (*n* = 5)	-	0.98 (0.04)	

The number of participants with data from the different sub-classes is indicated between brackets. Annotations for “Significant upper limb activity” and “Periodic” were only available for the 8 PD patients with tremor.

Activities in the control group that were sometimes still misclassified as tremor included brushing teeth, washing the hands or dishes, stirring in a cup of tea, nodding and typing. In one control participant, a 3 Hz tremor was observed while the participant was holding a spoon. In addition, some 4-sec predicted tremor windows showed a 3–5 Hz oscillatory gyroscope signal, but were not accompanied by rhythmic, oscillatory movement visible on the video recording.

### Generalizability of performance to PPP

To assess the generalizability of the developed tremor detection algorithm to PPP, we visually inspected and annotated a sample of 7160 windows among 179 PD participants from the PPP (Table 2). Since all windows annotated as ‘doubt’ could be either tremor or non-tremor, we considered the two extreme scenarios (all doubt labels are considered ‘annotated tremor’ or ‘annotated non-tremor’) to obtain a performance range. The positive predictive value was 0.80–0.94, and the negative predictive value was 0.88–0.96. Taking into account the prevalence of predicted tremor and non-tremor in each subject, the average sensitivity of the tremor detector on the PPP dataset was 0.37–0.58, and the average specificity was 0.98–1. When only considering participants with clinically observed rest tremor (MDS-UPDRS 3.17 ≥ 1), the average sensitivity was 0.44–0.64 and the average specificity was 0.98–0.99 (Supplementary Table 2), which is comparable to the performance in PD@Home.

**Table 2:** Annotations based on visual inspection of a sample of predicted tremor and predicted non-tremor windows among 179 PD participants from the PPP

	Predicted tremor	Predicted non-tremor
Annotated tremor	1622	625
Annotated non-tremor	67	3954
Annotated doubt	232	660
**Total**	**1921**	**5239**

### Construct validity of weekly tremor measures

From the amount of tremor windows detected during daytime (08:00 am–10:00 pm) and the tremor power per window, we derived four weekly aggregated tremor measures: tremor time, median tremor power, modal tremor power, and 90^th^ percentile of tremor power. Tremor time reflects the number of detected tremor windows while the arm was at rest or in a stable posture (expressed as percentage of inactive time). The median and modal tremor power represent the typical tremor severity, whereas the 90^th^ percentile of tremor power captures the maximal tremor severity.

**Fig. 2:**
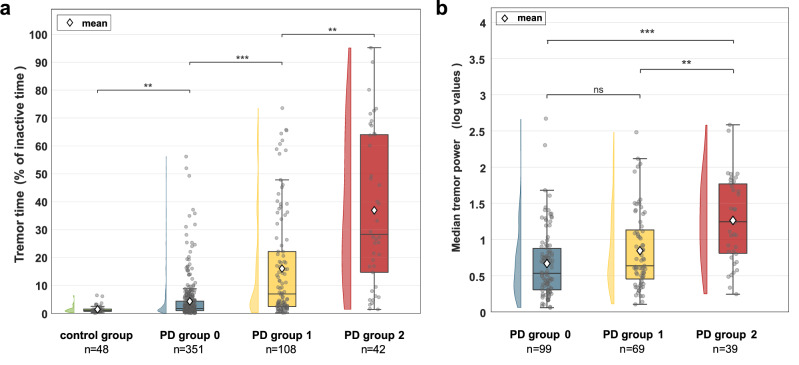
Group differences in weekly tremor measures. Three groups of PD participants with different clinical tremor severity were used (groups 0, 1 and 2 with MDS-UPDRS 3.17 of 0, 1 and ≥2 assessed in ON motor state in the device-sided arm). Non-PD controls were added for tremor time. **a** Tremor time was calculated as the number of detected tremor windows divided by all windows without non-tremor arm movements (inactive time) during daytime (08:00 am–10:00 pm), and expressed as percentage. **b** Median tremor power was calculated across all detected tremor windows during daytime, but only assessed if the tremor time was ≥ 3.5%. For both measures, the first week of collected data of PPP was used. The number of subjects in each subgroup is indicated. Significant differences (using Dunn’s test with Bonferroni correction) between subsequent groups are shown (*p < 0.05, ** *p* < 0.01, ***p < 0.001).

The construct validity of weekly tremor measures was assessed in three ways. First, we compared the weekly tremor measures between three groups of PD subjects with different clinical tremor severity ratings (MDS-UPDRS 3.17 ON) of the device-sided arm. Tremor time increased across these three groups of PD subjects, and was larger in PD group 0 compared to the control group (see Fig. 2a, using Dunn’s test with Bonferroni correction). Figure 2b shows the distribution of median tremor power across the different PD groups. The median tremor power in PD group 2 was larger than in PD group 0 and 1, but PD group 0 and 1 did not differ significantly. Group differences for the mode and 90^th^ percentile of tremor power are visualized in Supplementary Fig. 2. When using the MDS-UPDRS 3.17 score assessed in OFF instead of ON to stratify PD subjects, all group comparisons for the four weekly tremor measures were significantly different (Supplementary Fig. 3). Tremor power measures were only derived when the amount of tremor detected exceeded the false positive threshold of 3.5% (90^th^ percentile of tremor time detected in non-PD controls). Decreasing this threshold did not change the statistical difference in tremor power measures between participants with no/mild tremor and moderate/severe tremor (Supplementary Table 3). Groups 0 and 1 became more similar as the threshold was decreased, which was expected due to a larger number of participants with only false positives being detected in these groups.

**Fig. 3:**
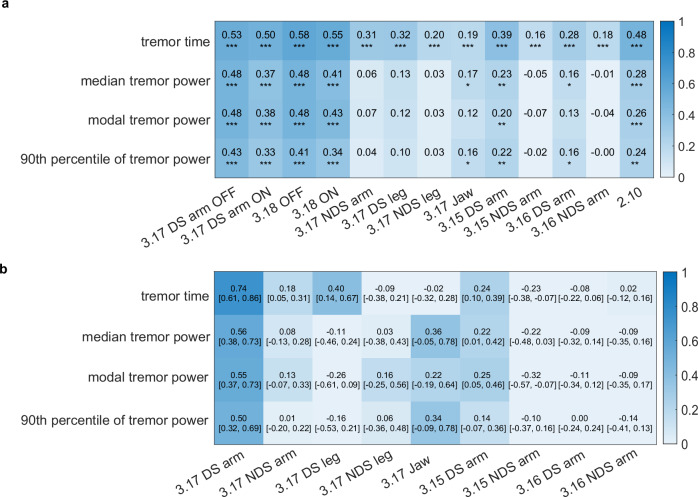
Univariable correlation and multivariable linear regression of weekly tremor measures with clinical tremor scores. In **a** Spearman’s correlation coefficients are shown with their significance level (*p < 0.05, **p < 0.01, ***p < 0.001, corrected using the false discovery rate method). Correlations with the rest tremor severity (3.17) and constancy (3.18) scores in the device-sided arm were assessed for ON and OFF scores separately. The other scores were averaged over ON and OFF motor states. In **b** the beta coefficients of the multivariable linear regression are shown with their 95% confidence intervals. Here, each weekly tremor measure was used as outcome and MDS-UPDRS tremor scores (averaged over ON and OFF conditions) as independent predictors. Beta coefficients were standardized by dividing them by the standard deviation of the weekly tremor measure, so that they represent the expected change in the measure (in standardized units) due to an increase in each MDS-UPDRS score of 1, with all other scores unchanged. The rest tremor constancy score (3.18) and patient-reported tremor score (2.10) were not included in **b** since these were not assessed for each body part separately. 3.15 = postural tremor severity, 3.16 = kinetic tremor severity, 3.17 = rest tremor severity, 3.18 = rest tremor constancy, DS device-sided, NDS non-device-sided.

We then assessed the association between the weekly tremor measures and clinical severity ratings of different tremor types in different body parts using univariable correlations (Spearman’s rank correlation) and multivariable linear regression (Fig. 3). Weekly tremor time correlated positively with all MDS-UPDRS part III tremor scores and with the patient-reported tremor score of MDS-UPDRS part II. Correlation was highest with the rest tremor constancy score (MDS-UPDRS 3.18) and rest tremor severity score (MDS-UPDRS 3.17) in the device-sided arm, followed by the patient-reported tremor score (MDS-UPDRS 2.10) and postural tremor severity score in the device-sided arm (MDS-UPDRS 3.15). The weekly tremor power measures also positively correlated with these clinical scores. To assess the added value of filtering out non-tremor arm movements, we also assessed correlations without filtering (Supplementary Fig. 4). For tremor time, the correlation with the postural and kinetic tremor severity scores in the device-sided arm increased without filtering (*p* < 0.01 for MDS-UPDRS 3.15 and *p* < 0.05 for MDS-UPDRS 3.16; using William’s test, corrected using the false discovery rate method). Furthermore, the correlation between median tremor power and the tremor constancy scores in OFF and ON decreased (*p* < 0.05). The correlation between the 90^th^ percentile of tremor power and both the clinical rest tremor severity (*p* < 0.05) and constancy scores (*p* < 0.01) in OFF and ON decreased without filtering. The other correlations did not significantly change.

**Fig. 4:**
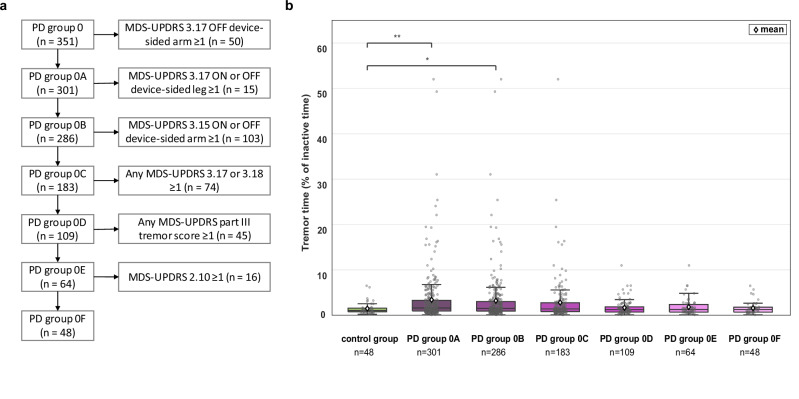
Tremor time in PD participants without clinically observed rest tremor in the device-sided arm. **a** Flowchart showing the incremental exclusion of subjects with clinical tremor scores ≥1 from PD group 0 (with MDS-UPDRS 3.17 ON of 0 in the device-sided arm). The order was based on the items’ predictive value for weekly tremor time in the multivariable regression. **b** Tremor time (as percentage of inactive time) measured in the first week of data collection of PPP in controls and different subgroups of PD group 0. The number of subjects in each subgroup is indicated. Significant differences (using Dunnett’s test) with the control group are shown (*p < 0.05, **p < 0.01, ***p < 0.001).

As expected, multivariable linear regression showed that rest tremor severity (MDS-UPDRS 3.17) in the device-sided arm was the strongest predictor for all weekly tremor measures (Fig. 3b). Rest tremor severity in the non-device-sided arm and device-sided leg, and postural tremor severity (MDS-UPDRS 3.15) in the device-sided arm were also significant predictors of weekly tremor time. To investigate why tremor time in PD group 0 (with clinically no rest tremor in the device-sided arm) was larger than in the control group, we systematically assessed the weekly tremor time in subgroups by incrementally excluding subjects from PD group 0 (Fig. 4). After excluding subjects with a rest tremor severity score (MDS-UPDRS 3.17) ≥ 1 in the device-sided leg and subjects with a postural tremor severity score (MDS-UPDRS 3.15) ≥ 1 in the device-sided arm, the subgroups did not differ significantly from the control group.

### Test-retest reliability of weekly tremor measures

In 495 PD subjects, the first and second week of data collection were valid and therefore used to assess the test-retest reliability of weekly tremor time. Tremor power measures were computed for the first two weeks in 187 PD subjects with at least 3.5% tremor time in both weeks. Excellent test-retest reliability was found for all weekly tremor measures, with ICC for tremor time of 0.98 (95%-CI 0.97–0.98), ICC for median tremor power of 0.96 (95%-CI 0.95–0.97), ICC for modal tremor power of 0.94 (95%-CI 0.92–0.95) and ICC for 90^th^ percentile of tremor power of 0.95 (95%-CI 0.93–0.96).

## Discussion

We developed and validated a generalizable algorithm for real-life monitoring of rest tremor in persons with PD, using a unique combination of two independent and complementary datasets: a small, but extensively video-labeled dataset at home, and a large, unsupervised dataset. By training a relatively simple logistic regression algorithm for tremor detection based on scale-insensitive MFCCs, we achieved generalizable performance when applied to a new dataset using a different wrist-worn device. This approach facilitates the use of our algorithm on different datasets and devices. In addition, we showed that weekly aggregated measures for tremor time and tremor power are reliable and clinically valid.

Our tremor detection algorithm had a sensitivity of 0.61 and specificity of 0.97 on the PD@Home data. On the PPP data we found a comparable performance in the group with clinically observed rest tremor in the device-sided arm. Furthermore, the amount of tremor detected in non-PD controls of PPP aligned with the specificity found in non-tremor PD participants and non-PD controls of PD@Home. The generalizability of published tremor detection algorithms is often unknown. One study found a decrease in specificity from 94.2% to 89.8% when applying a previously published tremor detection algorithm to their own collected dataset^21^, showing the importance of external validation. We found a slightly decreased sensitivity and increased specificity in the group without clinically observed rest tremor in the device-sided arm in PPP. A possible explanation is that our annotations based on visual inspection of gyroscope signals were more sensitive for tremor than the video-based annotations. Missed subtle tremor on the video recordings from PD@Home could also explain the decreased specificity in the tremor PD participants compared to the non-tremor PD participants and non-PD controls (i.e., some of the false-positives in the tremor PD group might have been actual tremor which was missed). Comparison to the performance of other tremor detection algorithms described in the literature is difficult due to variability in data collection across studies, mostly including standardized activities which differ from the unscripted daily life activities that we used^14–26^. In addition, our main aim was to obtain generalizable performance across different studies, rather than maximizing performance on a single dataset.

For its application in continuous, real-life monitoring of tremor, we prioritized high specificity, even at the expense of a slight decrease in sensitivity. A high specificity will limit the amount of false positives, which is important for continuous monitoring given the significant amount of time without tremor. Although our trained classifier combined with filtering by a clinical criterion achieved high specificity, it may not be feasible to eliminate all false positives using gyroscope signals, because we observed several rhythmic activities that closely resembled tremor (examples included e.g. brushing teeth, stirring in a cup of tea, nodding). Besides, the wrist-worn gyroscope sensor may detect subtle physiologic tremor in the forearm. Physiologic tremor is often described as a 8–12 Hz tremor, but its frequency depends on the mechanical characteristics of the joint^36^. The frequency of the unexplained rhythmic, oscillatory signals that were detected as tremor in the control group of PD@Home corresponded to normal forearm tremor at 3–5 Hz^36^.

Our weekly tremor measures showed excellent test-retest reliability, with ICC values similar or larger to those previously obtained for the MDS-UPDRS part III total score (ICC of 0.93)^37^. Noteworthy, items related to rest tremor severity (MDS-UPDRS 3.17) showed lower reliability^37^. Future longitudinal analyses could allow for a direct comparison of sensitivity to change of sensor and MDS-UPDRS based measures.

The weekly tremor measures were able to distinguish between non-PD controls and PD groups with different clinical rest tremor severity. Tremor time observed in PD subjects with a clinical rest tremor severity score of 0 (in both ON and OFF conditions) in their device-sided arm was larger than in controls, suggesting that remote monitoring may offer improved sensitivity for measuring PD tremor compared to clinical assessments in the device-sided arm. After excluding subjects with clinically observed rest tremor in the device-sided leg or postural tremor in the device-sided arm, the subgroup did not differ significantly from the control group regarding tremor time. We believe that a likely explanation is that participants with rest tremor in their device-sided leg have rest tremor in their device-sided arm as well (perhaps subtle and subclinical), which was not observed during the brief clinical assessment. Another possible explanation is that rest tremor in other limbs was detected at the wrist, for example when the device-sided arm was resting on the device-sided leg. The association between the clinical evaluation of postural tremor and the measured tremor time can likely be explained by the presence of re-emergent tremor in the device-sided arm, which is a continuation of rest tremor during stable posturing with a similar frequency^38^.

The moderate correlation of weekly tremor measures with MDS-UPDRS part III scores is in line with previous studies that continuously monitored PD tremor for more than 24 h^14,22,23^. Similar findings have been obtained in essential tremor^39^. At first glance, this may be viewed as a limitation of our algorithm, but in fact it has been shown that clinical assessments of PD tremor are very variable^8^, in line with the waxing and waning nature of PD tremor^40^, as well as its strong sensitivity to stress^41,42^. Hence, the primary goal of tremor detection algorithms should not be to maximize the correlation with an imperfect measure (i.e., the MDS-UPDRS, which provides only a snapshot in a very specific setting that is prone to observer bias)^43^. In fact, this raises the question which approach should become the gold standard against which to compare future tremor outcomes: the subjective and episodic clinically based score, or the objective score obtained at home from a digital device.

Our weekly tremor measures also moderately correlated with the patient-reported tremor score (2.10), with the highest correlation for tremor time. This suggests that the algorithm is able to give an impression of tremor burden in real life, despite the observed limited sensitivity for tremor. Understanding how sensor-derived tremor measures relate to experienced tremor burden by patients is an important prerequisite for implementing remote tremor monitoring in clinical care, as well as for obtaining regulatory approval to use the sensor-derived tremor measures as primary or secondary endpoint in clinical trials^28^.

There are some limitations to this study. First, the number of PD@Home participants with tremor was limited (*n* = 8), causing the estimated sensitivity of the tremor detector to be less precise. This was partially offset by the large number of participants in the second dataset where we replicated the findings. Second, the PPP dataset did not contain gold-standard tremor labels based on video-recordings. However, visual inspection of gyroscope signals was considered adequate to show that the performance on PD@Home was generalizable to PPP. Future research could elaborate on this by validating our algorithm on other external datasets and sensor devices. Third, by using only a single wrist-worn sensor, we focused on measuring tremor in the arm, although tremor in the legs and jaw may also occur. Besides, a wrist-worn sensor might not be able to detect subtle tremor in the fingers, limiting the sensitivity for detecting tremor. Nevertheless, including more sensors comes at the cost of decreasing compliance, and tremor in the arm is the most prevalent type of tremor in PD^44^. Our prior work in the PPP cohort showed that long-term compliance (over a 2-year time frame) with using a wrist-worn device is excellent, with a median wear time of 21 h per day, and a drop-out rate of just 5.4%^8^. This facilitates long-term continuous monitoring of tremor in real life.

Real-life monitoring of PD rest tremor with our developed tremor detector could benefit both patient care and clinical trials. By averaging over larger time periods in patients’ own environments, sensor-based tremor quantification provides a solution to the problem that hospital-based clinical tremor ratings are often not representative of the actual PD tremor severity^8^. Clinical trials may benefit from the potentially improved sensitivity of objective tremor measures compared to current clinical scales, because this will reduce the required sample size and costs^28^. However, before objective tremor measures can be implemented as outcome measures in randomized controlled trials, it will be necessary to determine their sensitivity to detect a clinically relevant change^45^. Besides, sensor-based tremor assessments over longer periods of time may provide more insight into clinically relevant tremor characteristics at the individual level, such as the response to (dopaminergic) medication, the effect of stress on tremor in daily life, and change in tremor severity over time. This could all help in developing personalized treatments and ultimately improving the quality of life of persons with PD.

## Methods

### Datasets

The Parkinson@Home Validation study was used to train the tremor detector. Details concerning the data collection procedure are described elsewhere^33^. Briefly, 25 persons with PD and 25 age-matched non-PD controls were visited at their own homes and recorded on video for at least one hour while they performed unscripted daily life activities, before and after intake of dopaminergic medication for PD participants. Simultaneously, sensor data was collected at several body locations. For this study, we used the gyroscope data collected with the wrist-worn Physilog 4 device at a sampling rate of 200 Hz. The advantage of using gyroscope data over accelerometer data is that filtering out the gravitational component is not needed^46^. For PD participants with tremor, the sensor at the side with most severe tremor was chosen. We selected sides to match for hand dominance for the other participants. We excluded two subjects due to technical issues with the sensor devices, yielding a training dataset of 24 PD participants and 24 controls (for demographic and clinical characteristics see Supplementary Table 4).

Based on the concurrent video recordings, the presence and severity of tremor during the unscripted activities were annotated by trained research assistants, and checked by a neurologist. The annotations included any type of tremor observed, because the video recordings during daily life activities were not deemed suitable to reliably distinguish between different tremor subtypes. The video annotation protocol can be found in Supplementary Fig. 5. In 8 PD patients, tremor was observed during the unscripted daily life activities, whereas in the other 16 PD patients, no tremor was observed. Because of the low prevalence of moderate and severe tremor in the dataset, we focused on detecting only the presence of tremor. The median amount of annotated tremor used for training the tremor detector was 14 min (inter-quartile range (IQR) 7–36 min across the eight PD participants with tremor in PD@Home), and the median amount of annotated non-tremor was 109 min (IQR 86–145 min across all 48 participants in PD@Home). For tremulous PD participants, significant upper limb activities and periodic activities that could resemble tremor were annotated as well. The presence of the following activities was annotated for all participants: sitting, standing, gait, postural transitions, running or exercising, cycling and driving a motorized vehicle.

The trained tremor detector was subsequently applied to unsupervised gyroscope data collected with the Verily Study Watch during the Personalized Parkinson Project^34^. 520 early-stage PD participants were asked to wear the watch preferably 24/7 for 2 years, except during charging (approximately 1 h per day), at their preferred side. In addition, 50 non-PD controls were asked to wear the same watch for 1 year. The Verily Study Watch typically had a sampling frequency 100 Hz, although a lower sampling frequency of 50 Hz occurred in some participants at the beginning of the study. In this study, we used data collected during the first 3 weeks of follow-up. For three patients, no sensor data was collected, yielding a dataset of 517 patients and 50 controls. Demographic and clinical information, including MDS-UPDRS part II and III scores in OFF and ON motor conditions for PD subjects, was collected at baseline (Supplementary Table 5).

The PD@Home and PPP studies were both conducted in accordance with the Declaration of Helsinki and Good Clinical Practice guidelines, and were approved by the local medical ethics committee (Commissie Mensgebonden Onderzoek, regio Arnhem-Nijmegen, reference number 2015–1776 for the PD@Home study and reference number 2016–2934 for the PPP). All participants provided informed consent prior to enrollment.

### Preprocessing, feature extraction and tremor detection

Gyroscope signals were down-sampled to 50 or 100 Hz, depending on the closest original sampling frequency. Based on 4-s windows, we obtained the power spectral density (PSD) of each gyroscope axis signal (using Welch’s method). We then summed the PSDs across the three axes and extracted the peak frequency and 12 mel-frequency cepstral coefficients (MFCCs) from this. MFCCs were computed using 15 filterbanks in the range of 0–25 Hz with filter edges defined by the adjusted mel-scale for inertial signals^47^. MFCCs have been popular in audio signal analysis because they capture the overall shape of the spectral envelope with a small, uncorrelated and scale-invariant feature set. The scale-invariance of MFCCs is important for the generalizability of the tremor detection algorithm and its robustness to gyroscope drift. Earlier work on tremor detection algorithms based on MFCCs showed promising results^26^.

Based on the video annotations, windows extracted from the PD@Home dataset were labeled as tremor if this was present for at least 50% of window time. The most prevalent type of activity was chosen as the activity label. A logistic regression classifier was trained to detect tremor based on the MFCCs, with the threshold set at 95% specificity. During training, windows labeled as cycling were oversampled by a factor of 100 to penalize the algorithm for detecting cycling as tremor. In the PD@Home dataset, the MFCCs outperformed simple PSD-based features (Supplementary Fig. 6).

We aimed to detect rest and re-emergent tremor with high specificity. Therefore, an additional clinical criterion was set for detecting tremor: the peak frequency must be within the rest tremor frequency range of 3-7 Hz^7^. Furthermore, we used a rule-based non-tremor arm movements detector to filter out windows with simultaneous non-tremor arm movements. The aim of this was (1) to further improve the specificity for rest tremor, and (2) to improve the accuracy of measured tremor power. If rest tremor and non-tremor arm movements are simultaneously present (e.g. tremor during gait with arm swing), the tremor peak in the power spectral density could be distorted by the higher harmonics of the slower movement (Supplementary Fig. 7). Gyroscope power within the 0.5–3 Hz band was used to distinguish slow voluntary movements from higher frequency tremor and lower frequency gyroscope drift. We visualized the distribution of this feature across all windows of the PD@Home dataset. The threshold for non-tremor arm movements was selected based on K-means clustering with two clusters (Supplementary Fig. 8).

### Evaluation of tremor detection performance

First, the tremor detection algorithm was evaluated on the PD@Home dataset using Leave-One-Subject-Out Cross-Validation (LOSO-CV), i.e., we evaluated its sensitivity and specificity on one subject, after training on all other subjects. In addition to the overall performance, we compared the specificity stratified for different annotated activities. Video recordings of detected tremor windows in the control group were visually inspected to identify other activities that resemble tremor.

Next, we assessed the generalizability of the developed tremor detection algorithm to PPP as the external dataset. For this dataset no video recordings were available, but due to the rhythmic, oscillatory movement at a characteristic frequency of 3–7 Hz, PD rest tremor can be distinguished from other movements based on visual inspection of the gyroscope signals. We visually inspected and annotated a sample of 7160 windows, sampled from the third week of data collection in 179 PD participants. Based on an earlier version of our tremor detection algorithm, for each subject we randomly selected 20 predicted tremor and 20 predicted non-tremor windows between 7:00 am and 11:00 pm in the third week of data collection. The ratio between predicted tremor and non-tremor windows in the final dataset changed from 1:1 to approximately 1:3, because of further improvements of the tremor detection algorithm to increase its specificity based on PD@Home (i.e., the addition of an extra criterion for rest tremor and filtering out windows with non-tremor arm movements). After visual inspection of the raw gyroscope signals and PSDs, we annotated the windows as tremor, non-tremor or doubt (the annotation protocol is given in Supplementary Fig. 9). The first 1800 windows were annotated by the first author of this paper and by a second annotator. Windows for which there was disagreement or doubt were discussed. The inter-rater agreement was substantial (Cohen’s kappa of 0.73), therefore only the first author of this paper annotated the remaining windows. The signal-based tremor labels were used to estimate the sensitivity and specificity for PPP, which were then compared to the performance on PD@Home.

To evaluate the effect of tremor power on algorithm performance, we also stratified the subjects across their clinical rest tremor severity score (MDS-UPDRS 3.17 OFF) at the device-sided arm (60 subjects had a score of 0, 60 had a score of 1 and 59 had a score of ≥2). The effect of other PD motor symptoms on algorithm performance was assessed by splitting the subjects into two other subgroups based on the total UPDRS part III score minus the total tremor score (for the first group this score was <28 and for the second group ≥28).

### Weekly tremor measures

After we obtained window-level tremor predictions for the first two weeks of PPP data, we quantified tremor time and power based on data collected during daytime (08:00 am–10:00 pm). These tremor measures were aggregated over one-week periods, to enable assessment of longitudinal progression in future research. Only valid weeks were considered, which were defined, based on previous research, as weeks with at least three valid days with ≥10 h of sensor data^48^.

For each valid week, tremor time was calculated as the number of detected tremor windows divided by the number of windows without detected non-tremor arm movements (inactive time) during valid days. Tremor power was first calculated for each detected tremor window by ${\log }_{10}({P}_{T}+1)$, where ${P}_{T}$ is the power within a bandwidth of 1.25 Hz around the dominant frequency in the tremor frequency band of 3–7 Hz^49^. The logarithm was taken because of the linear relationship between clinical tremor severity ratings and the log of tremor displacement or angular velocity^50^. We added a constant of 1 before taking the logarithm to make sure that ${P}_{T}$ of 0 corresponds to a tremor power of 0. Then we derived three weekly aggregated tremor power measures: the median, mode (i.e., the peak in the probability density function of tremor power) and 90^th^ percentile of tremor power.

In the first week, a median of 75.1 h (IQR 65.4–78.0 h) of gyroscope data collected during daytime in 501/517 PD participants was used for analysis. For controls, this was a median of 75.6 h (IQR 65.5–80.4 h) in 48/50 subjects. The median percentage of time with non-tremor arm movements was 45.1% (IQR 37.4%–53.1%) in the PD group and 50.2% (IQR 44.3%–59.3%) in the control group (for more details see Supplementary Table 6). Weekly tremor power measures were only derived for the PD group, and only if aggregated tremor time exceeded 3.5%, which held for 207/517 PD participants in the first week. The threshold of 3.5% was based on the 90^th^ percentile of tremor time detected in non-PD controls, and used to avoid inaccurate estimates due to a large false positive fraction of all detected tremor windows.

### Construct validity and test-retest reliability

The construct validity of weekly tremor measures was assessed in three ways. First, we compared the weekly tremor measures between three groups of PD subjects with increasing rest tremor severity scores (MDS-UPDRS 3.17 ON) of their device-sided arm. PD group 0 had a score of 0 (no tremor; *n* = 351), group 1 had a score of 1 (slight tremor, *n* = 108), and group 2 had a score of ≥ 2 (mild to severe tremor; *n* = 42). Group 2 contained a wider range of clinical tremor severities (MDS-UPDRS scores of 2, 3, or 4), because the number of participants for each of these scores was relatively low. We used the scores assessed in ON motor condition for participants on anti-parkinsonian medication, because we expected that this best reflects their condition in daily life. Weekly tremor time was also quantified in non-PD controls of PPP.

Second, we assessed the contribution of different tremor types in different body parts to the weekly tremor measures by univariable correlations (Spearman’s rank correlation) and by multivariable linear regression, hypothesizing that the weekly tremor measures are predominantly associated with the rest tremor severity in the device-sided arm. Third, we further investigated the potential causes of tremor detected in PD subjects with an MDS-UPDRS 3.17 ON score in the device-sided arm of 0, by incrementally excluding subjects with other tremor scores of 1 or larger.

Finally, the test-retest reliability of all weekly tremor measures was assessed by the Intraclass Correlation Coefficient (ICC) between the first two weeks in PD subjects of the PPP dataset^51^.

## Supplementary information

Supplementary information

## Data Availability

The data used to train the tremor detector on the Parkinson@Home dataset is publicly available in the Radboud Data Repository: 10.34973/2xxa-g520. Access to the full PD@Home Validation study - with the exception of the raw video recordings - can be arranged through a request to the Michael J Fox Foundation (www.michaeljfox.org). Data from the Personalized Parkinson Project is available on reasonable request via: https://www.personalizedparkinsonproject.com/home/data/requesting.

## Code availability

The code used to train and validate the tremor detector on PD@Home is publicly available in the Git repository: https://github.com/biomarkersParkinson/pdathome_tremor. An implementation of the tremor detection algorithm, used to generate the results in this study on the PPP dataset, is available as part of the ParaDigMa python toolbox (10.5281/zenodo.15223364).
