# Supplementary material for: A generalizable and open-source algorithm for real-life monitoring of tremor in Parkinson’s disease
Source: NPJ Parkinsons Dis. 2025 Jul 10;11:205. doi: 10.1038/s41531-025-01056-2 (PMC12246068; doi:10.1038/s41531-025-01056-2)
Supplement: Supplementary file 1 — Supplementary information [file 41531_2025_1056_MOESM1_ESM.pdf]

Supplementary tables

Supplementary Table 1: Tremor detection performance on PD@Home (average across leave-one-subject out cross-validation folds, and standard deviation between brackets), without filtering out windows with detected non-tremor arm movements. The number of participants with data from the different sub-classes is indicated between brackets. Annotations for "Significant upper limb activity" and "Periodic" were only available for the 8 PD patients with tremor.

	**Sensitivity**	**Specificity**	**Weighted specificity across sitting, standing, gait and postural transitions**
Overall (n=48)	0.70 (0.18)	0.96 (0.05)	
**Subgroup**			
Tremor PD (n=8)	0.70 (0.18)	0.90 (0.04)	0.90 (0.05)
Non-tremor PD (n=16)	-	0.96 (0.06)	0.96 (0.05)
Non-PD controls (n=24)	-	0.98 (0.04)	0.98 (0.01)
**Type of activity**			
Sitting (n=8/n=48)	0.70 (0.25)	0.95 (0.07)	
Standing (n=7/n=48)	0.66 (0.31)	0.97 (0.03)	
Gait (n=7/n=48)	0.39 (0.27)	0.98 (0.04)	-
Postural transitions (n=48)	-	0.99 (0.03)	
Running/Exercising (n=5)	-	1 (0)	
Cycling (n=14)	-	0.97 (0.08)	-
Driving motorized vehicle (n=2)	-	0.92 (0.10)	
Significant upper limb activity (n=8)	-	0.95 (0.06)	-
Suspicious activity (n=5)	-	0.83 (0.18)	-

Supplementary Table 2: Performance of the tremor detection algorithm on PPP based on the visual inspection and annotation of a sample of windows, for different subgroups of PD participants. The number of subjects in each subgroup is given.

**Subgroup**	**Sensitivity**	**Specificity**
MDS-UPDRS 3.17 OFF = 0 (n=60)	0.25 – 0.48	0.99 - 1
MDS-UPDRS 3.17 OFF = 1 (n=60)	0.41 – 0.65	0.99
MDS-UPDRS 3.17 OFF ≥ 2 (n=59)	0.46 – 0.62	0.97 – 0.99
MDS-UPDRS part 3 OFF – tremor subscore < 28 (n=89)	0.34 – 0.57	0.99 - 1
MDS-UPDRS part 3 OFF – tremor subscore ≥ 28 (n=90)	0.40 – 0.59	0.98 – 0.99
Overall (n=179)	0.37 – 0.58	0.98 – 1

Supplementary Table 3: p-values (using Dunn's test with Bonferroni correction) of differences in weekly aggregated tremor power measures between the three PD groups (groups 0, 1 and 2 with MDS-UPDRS 3.17 of 0, 1 and ≥2 assessed in OFF motor state in the device-sided arm), using different thresholds for selection of participants.

		**No threshold**	**> 2% tremor time**	**> 3.5% tremor time**	**> 5% tremor time**
**Median tremor power**	**PD group 0 vs 1**	p = 1.0	p < 0.05	p < 0.05	p < 0.01
	**PD group 0 vs 2**	p < 0.001	p < 0.001	p < 0.001	p < 0.001
	**PD group 1 vs 2**	p < 0.001	p < 0.001	p < 0.001	p < 0.01
**Modal tremor power**	**PD group 0 vs 1**	p = 1.0	p <0.01	p <0.01	p <0.01
	**PD group 0 vs 2**	p < 0.001	p < 0.001	p < 0.001	p < 0.001
	**PD group 1 vs 2**	p < 0.001	p < 0.001	p < 0.001	p < 0.001
**90th percentile of tremor power**	**PD group 0 vs 1**	p = 1.0	p = 0.23	p < 0.05	p < 0.05
	**PD group 0 vs 2**	p < 0.001	p < 0.001	p < 0.001	p < 0.001
	**PD group 1 vs 2**	p < 0.001	p < 0.001	p < 0.001	p < 0.001

*Supplementary Table 4: Demographic and clinical characteristics of PD participants and non-PD controls of the PD@Home dataset included in the analyses. IQR: inter-quartile range. MDS-UPDRS: Movement Disorder Society-Sponsored Revision of the Unified Parkinson's Disease Rating Scale. Part 1: non-motor experiences of daily living. Part 2: motor experiences of daily living. Part 3: motor examination. Part 4: motor complications. *: 1 missing value.*

		PD subjects with annotated tremor (n=8)	PD subjects with no annotated tremor (n=16)	Non-PD controls (n=24)
**Age (years), median (IQR)**		61.0 (58.3 - 69.0)	66.0 (61.0 - 70.5)	67.5 (55.0 - 70.0)
**Gender (men), n (%)**		4 (50%)	7 (44%)	13 (54%)
**Time since diagnosis of PD (years), median (IQR)**		7.0 (5.3 - 9.5)	7.0 (5.0 - 11.0)	-
**Hoehn and Yahr stage in off state, n (%)**				
Stage 1		1 (12.5%)	0 (0.0%)*	-
Stage 2		6 (75.0%)	10 (62.5%)	-
Stage 3		0 (0.0%)	4 (25.0%)	-
Stage 4		1 (12.5%)	1 (6.3%)	-
**MDS-UPDRS, median (IQR)**				
Part 1	(scale range: 0 to 52)	10.5 (7.0 - 17.3)	9.5 (8.0 - 15.0)	3.0 (0.3 - 4.0)
Part 2	(scale range: 0 to 52)	10.5 (8.3 - 14.5)	9.0 (7.3 - 13.0)	0.0 (0.0 - 0.0)*
Part 3 (off state)	(scale range: 0 to 132)	50.4 (38.8 - 61.8)	35.0 (30.0 - 46.8)	6.5 (4.3 - 11.0)
Part 3 (on state)	(scale range: 0 to 132)	32.0 (24.0 - 37.5)	25.5 (17.5 - 38.0)	-
Part 4	(scale range: 0 to 24)	6.0 (3.5 - 9.8)	6.0 (4.3 - 8.5)	-
**Tremor sub-score of MDS-UPDRS part III, median (IQR)**				
Off state	(scale range: 0 to 40)	14.0 (10.3 - 18.8)	4.0 (2.3 - 8.5)	0.5 (0.0 - 1.8)
On state	(scale range: 0 to 40)	9.5 (3.0 - 11.8)	2.0 (1.0 - 5.0)	-
**Rest tremor severity (arm of most affected side), n (%)**				
0: normal (off | on)		0 (0.0%) | 2 (25.0%)	14 (87.5%) | 15 (93.8%)	24 (100%)
1: slight (off | on)		1 (12.5%) | 2 (25.0%)	0 (0.0%) | 1 (6.3%)	0 (0.0%)
2: mild (off | on)		3 (37.5%) | 2 (25.0%)	1 (6.3%) | 0 (0.0%)	0 (0.0%)
3: moderate (off | on)		3 (37.5%) | 2 (25.0%)	1 (6.3%) | 0 (0.0%)	0 (0.0%)
4: severe (off | on)		1 (12.5%) | 0 (0.0%)	0 (0.0%) | 0 (0.0%)	0 (0.0%)

*Supplementary Table 5: Demographic and clinical characteristics of PD participants and non-PD controls of the PPP dataset included in the analyses. IQR: inter-quartile range. MDS-UPDRS: Movement Disorder Society-Sponsored Revision of the Unified Parkinson's Disease Rating Scale. Part 1: non-motor experiences of daily living. Part 2: motor experiences of daily living. Part 3: motor examination. Part 4: motor complications. *: 23 missing values. †: 21 missing values. ‡: 6 missing values. §: 32 missing values. ¶: 12 missing values. #: 26 missing values.*

		PD subjects (n=517)	Non-PD controls (n=50)
**Age (years), median (IQR)**		62 (55 - 69)	70 (66 – 74)
**Gender (men), n (%)**		305 (59%)	18 (36%)
**Time since diagnosis of PD (years), median (IQR)**		2.7 (1.4 - 3.9)	-
**Hoehn and Yahr stage in off state, n (%)**			
Stage 1		46 (8.9%)	-
Stage 2		406 (78.5%)	-
Stage 3		59 (11.4%)	-
Stage 4		6 (1.2%)	-
**MDS-UPDRS, median (IQR)**			
Part 1	(scale range: 0 to 52)	9 (7 - 14)*	-
Part 2	(scale range: 0 to 52)	7 (4 - 12)†	-
Part 3 (off state)	(scale range: 0 to 132)	32 (24 - 42)‡	-
Part 3 (on state)	(scale range: 0 to 132)	27 (19 - 36)§	-
Part 4	(scale range: 0 to 24)	1 (0 - 5)¶	-
**Tremor sub-score of MDS-UPDRS part III, median (IQR)**			
Off state	(scale range: 0 to 40)	4 (2 - 7)	-
On state	(scale range: 0 to 40)	3 (1 – 5.5)#	-
**Rest tremor severity (device-sided arm), n (%)**			
0: normal (off | on)		336 (65.0%) |353 (71.9%)	-
1: slight (off | on)		121 (23.4%) | 101 (20.6%)	-
2: mild (off | on)		37 (7.2%) | 27 (5.5%)	-
3: moderate (off | on)		23 (4.5%) | 10 (2.0%)	-
4: severe (off | on)		0 (0.0%) | 0 (0.0%)	-

Supplementary Table 6: Median (interquartile range) percentage of daytime with non-tremor arm movements and percentage of detected tremor windows removed by filtering out windows with non-tremor arm movements in the first week of collected data of PPP. PD participants were split in three groups: PD group 0 had an MDS-UPDRS 3.17 score of 0 in the device-sided arm in ON motor state, PD group 1 had a score of 1 and PD group 2 a score of ≥2.

Subgroup	Percentage of daytime with detected arm movements (%)	Percentage of detected tremor removed by filtering out windows with detected arm movements (%)
Non-PD controls	50 (44 – 59)	38 (27 – 53)
PD group 0	47 (39 – 54)	28 (16 – 42)
PD group 1	41 (35 – 49)	17 (7 – 30)
PD group 2	39 (30 – 46)	18 (8 – 25)

Supplementary figures

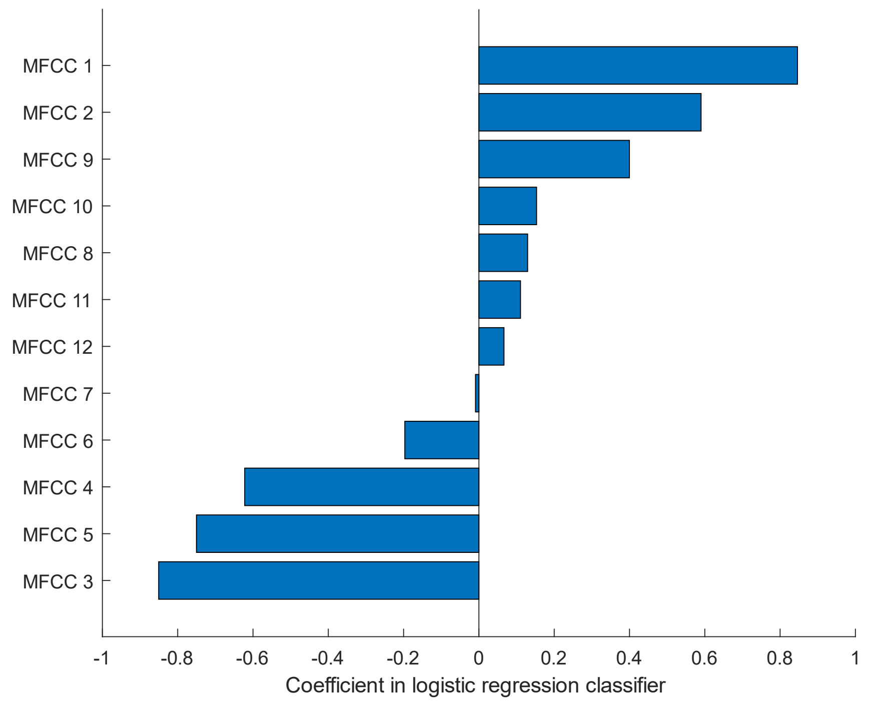

Supplementary Figure 1: Coefficients in the logistic regression classifier for the different mel-frequency cepstral coefficients (MFCCs).

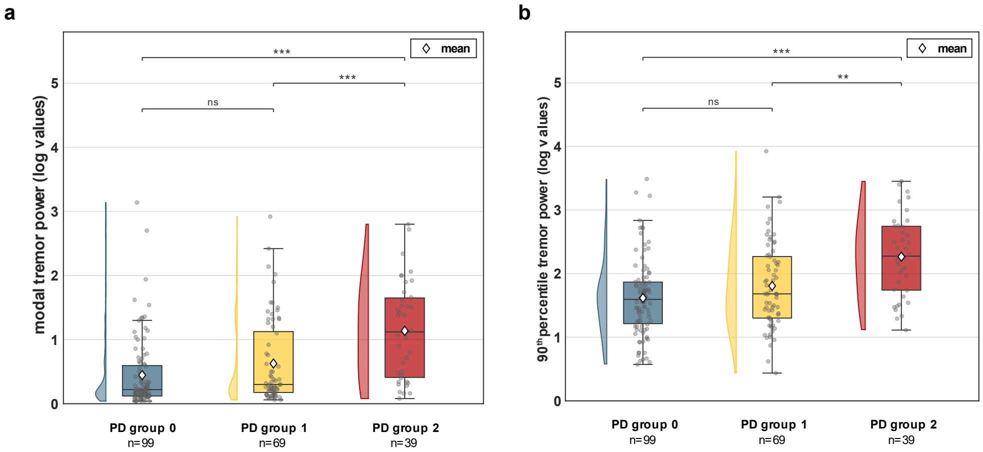

Supplementary Figure 2: Group differences in weekly tremor measures. a: *Modal tremor power and* ***b:*** *90th percentile of tremor power, measured in the first week of collected data of PPP in three groups of PD participants (groups 0, 1 and 2 with MDS-UPDRS 3.17 of 0, 1 and ≥2 assessed in ON motor state in the device-sided arm). Both measures were calculated across all detected tremor windows during daytime, but only assessed if the tremor time was ≥3.5%. The number of subjects in each subgroup is indicated. Significant differences (using Dunn's test with Bonferroni correction) between subsequent groups are shown (* p<0.05, ** p<0.01, *** p<0.001*

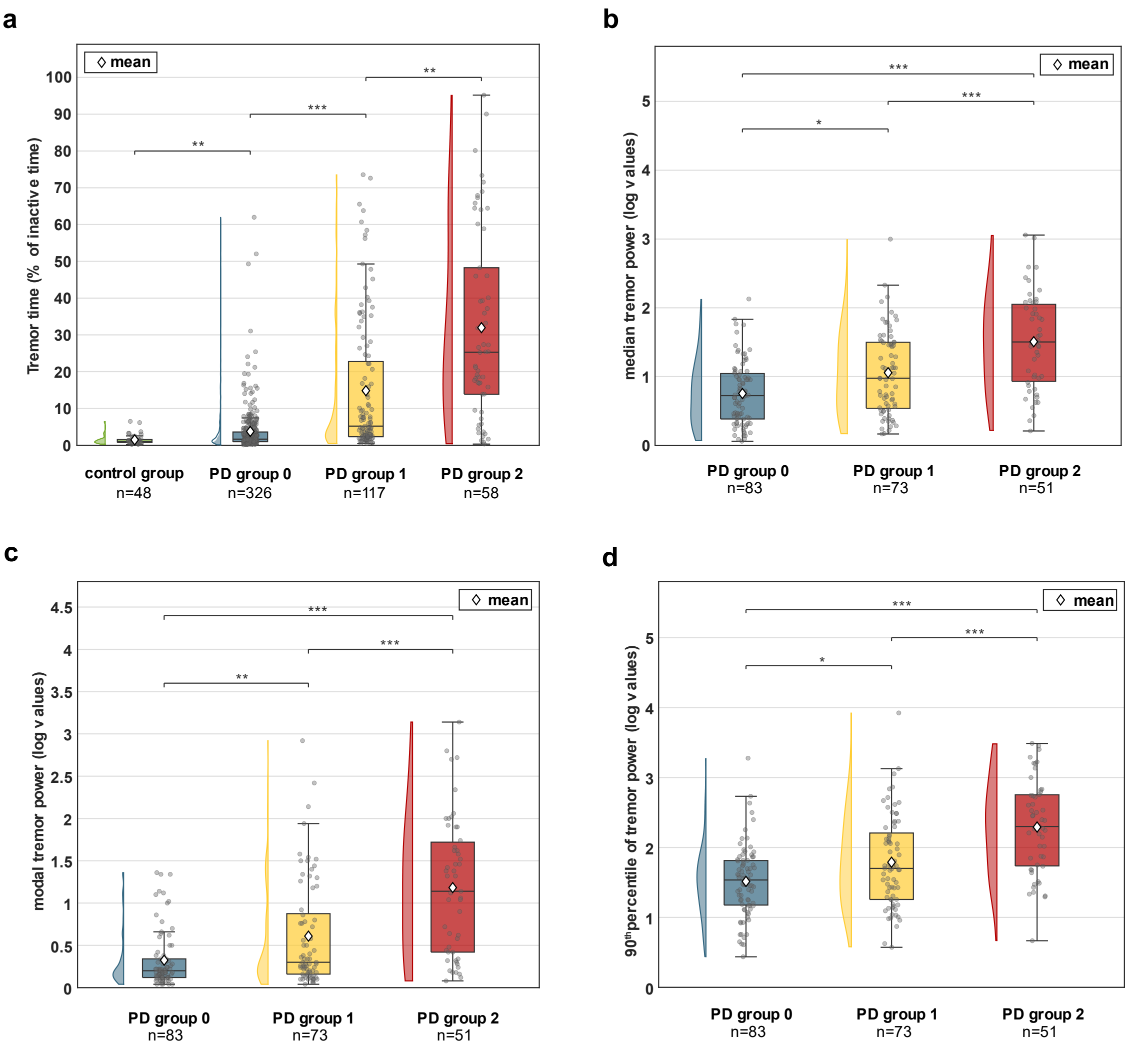

Supplementary Figure 3: Group differences in weekly tremor measures, based on clinical tremor severity scores assessed in OFF motor state *(groups 0, 1 and 2 with MDS-UPDRS 3.17 of 0, 1 and ≥2 assessed in OFF motor state in the device-sided arm). **a:** Tremor time was calculated as the number of detected tremor windows divided by all windows without detected non-tremor arm movements (inactive time) during daytime (08:00 am – 10:00 pm), and expressed as percentage. **b:** Median tremor power, **c:** modal tremor power and **d:** 90th percentile of tremor power were calculated across all detected tremor windows during daytime, but only assessed if the tremor time was ≥3.5%. For all measures, the first week of collected data of PPP was used. The number of subjects in each subgroup is indicated. Significant differences (using Dunn's test with Bonferroni correction) between subsequent groups are shown (* p<0.05, ** p<0.01, *** p<0.001).*

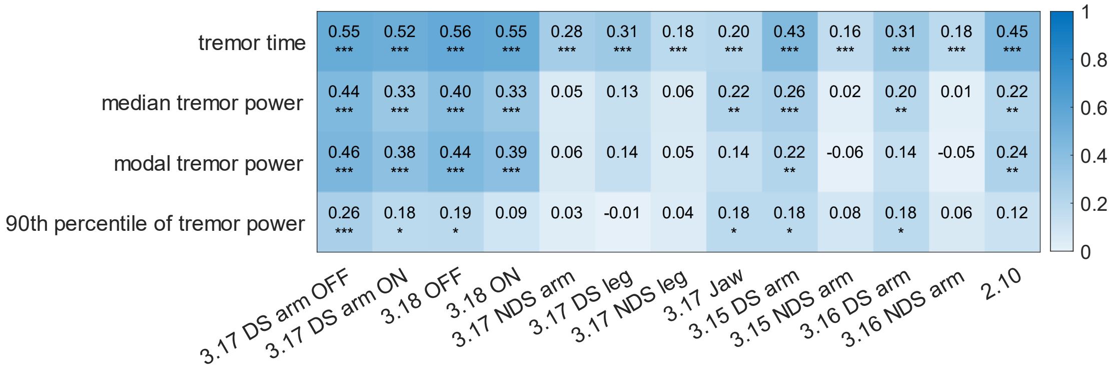

Supplementary Figure 4: Correlation of weekly tremor measures with clinical tremor scores, without filtering out the windows with detected non-tremor arm movements. *Spearman's correlation coefficients are shown with their significance level (* p<0.05, ** p<0.01, *** p<0.001, corrected using the false discovery rate method). Correlations with the rest tremor severity (3.17) and constancy (3.18) scores in the device-sided arm were assessed for ON and OFF scores separately. The other scores were averaged over ON and OFF motor states. 3.15 = postural tremor severity, 3.16 = kinetic tremor severity, 3.17 = rest tremor severity, 3.18 = rest tremor constancy, DS = device-sided, NDS = non-device-sided.*

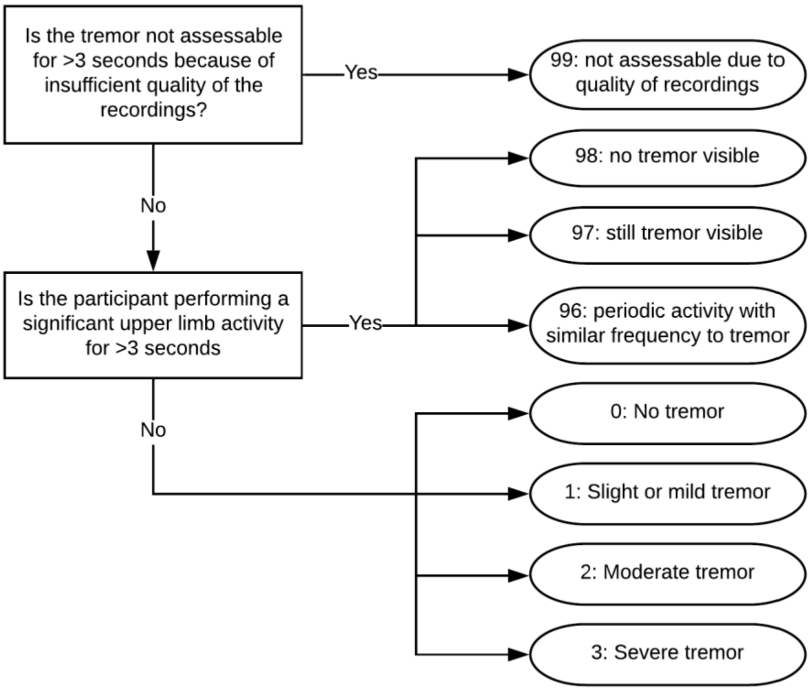

Supplementary Figure 5: Schematic overview of the video annotation protocol for the presence and severity of tremor used in the PD@Home study. *For training the tremor detector we only considered the presence of tremor (labels 97, 1, 2 and 3) or not (labels 98, 96 and 0).*

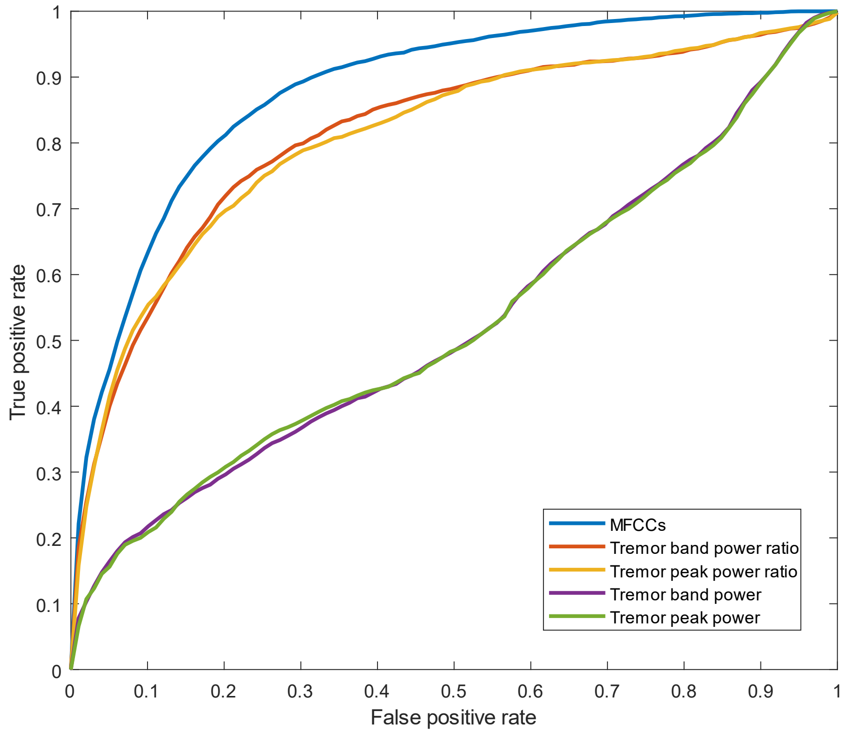

Supplementary Figure 6: Receiver operating characteristic (ROC) curves of the logistic regression tremor classifiers, *one based on mel-frequency cepstral coefficient (MFCCs), the other four trained with a simple Power Spectral Density based feature. Tremor band power was defined as the 3-7 Hz power. Tremor peak power was computed as the power 1.25 Hz around the tremor peak (dominant frequency in 3-7 Hz range). The tremor band power ratio and tremor peak power ratio were obtained by dividing the tremor band power and tremor peak power by the 0.5-25 Hz power. The ROC curves were averaged across the eight PD participants with tremor in the PD@Home dataset.*

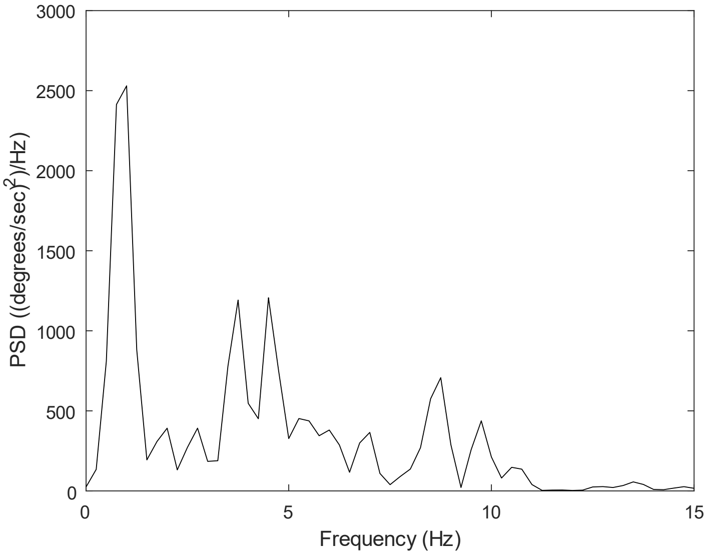

Supplementary Figure 7: Power spectral density (PSD) estimation of a 4-second gyroscope signal measured during tremor and gait in a subject of the Parkinson@Home dataset. *The PSD was summed over all three gyroscope axes. The 4-5 Hz tremor peak is distorted by the higher harmonics of gait.*

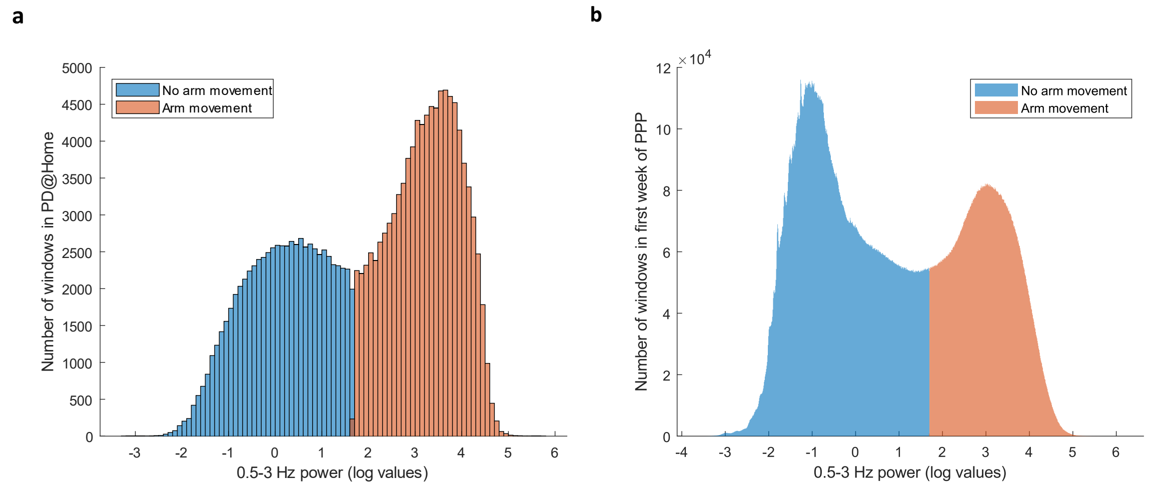

Supplementary Figure 8: Detection of non-tremor arm movements. a: *Distribution of the power in the 0.5-3 Hz band (log values) across all windows of PD@Home. The threshold for non-tremor arm movements was determined by K-means clustering with 2 clusters, yielding a threshold of 50 deg²/s². **b:** Distribution of the same feature across all windows collected during daytime in the first week of PPP. Using the same threshold, the proportion of windows with non-tremor arm movements is smaller than in PD@Home where people were encouraged to perform activities of daily living.*

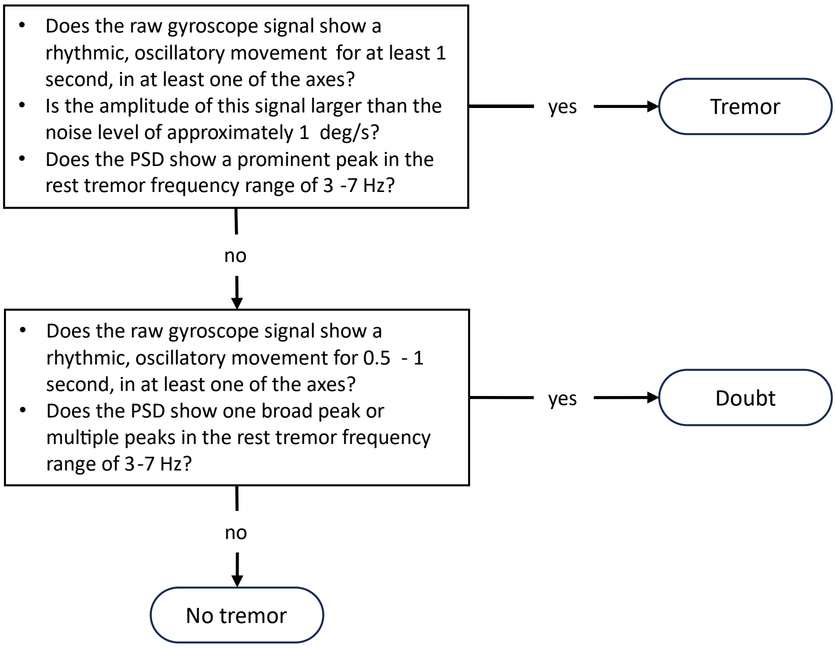

Supplementary Figure 9: Schematic overview of the annotation protocol for the presence of tremor in the PPP study, *based on visual inspection of 4-second gyroscope signals and power spectral density (PSD) estimations.*
